# De Clérambault’s syndrome revisited: a case report of Erotomania in a male

**DOI:** 10.1186/s12888-020-02921-5

**Published:** 2020-10-23

**Authors:** Maria Teresa Tavares Rodrigues Tomaz Valadas, Lucilia Eduarda Abrantes Bravo

**Affiliations:** Psychiatry Service, Department of Psychiatry and Mental Health, Local Health Unit of Lower Alentejo, Rua Dr. Antonio Fernando Covas Lima, 7801-849 Beja, Portugal

**Keywords:** Erotomania, de Clérambault’s syndrome, Delusional disorder, Male

## Abstract

**Background:**

Erotomania, also known as “de Clérambault’s Syndrome”, is a psychiatric syndrome characterized by the delusional belief that one is loved by another person of, generally of a higher social status. Erotomania has always been a target of attempts of conceptualization, and the utility of regarding Erotomania as an independent syndrome has been questioned to this day. Erotomania has a much higher prevalence in the female sex, and male Erotomania is a rare and probably underdiagnosed condition. Male Erotomania is only more prevalent in forensic samples, since male sex is one of the risk factors for violent behavior in this disorder. In this article, we aim to describe an uncommon case of Erotomania occurring in a male, hoping to add to literature and to reflect on the implications of the occurrence of Erotomania in men. By discussing the case in light of the different described clinical pictures, proposed diagnostic criteria and classifications, we also aim to contribute to the ongoing attempt to conceptualize this syndrome and to understand the pertinence of considering it an independent nosological entity.

**Case presentation:**

We describe a case of Erotomania in a middle-aged Caucasian Portuguese male, with consecutive erotic delusions, followed by a classic turn to a persecutory delusion. The patient was admitted as an inpatient in a psychiatry unit and he was medicated with risperidone 3 mg and diazepam 3 mg daily. His persecutory delusion remitted a 4 days later, he gained insight and was discharged to follow-up as an outpatient. He retains his erotic delusional beliefs, but these are less intense, and has not presented further aggressive behavior.

**Conclusions:**

We can conclude that it seems reasonable to retain the operative concept of Erotomania as a subtype of Persistent Delusional Disorder/Delusional Disorder, since cases fitting the classical descriptions of the syndrome have been reported, including the presented case. The diagnosis of Erotomania has implications in case management, treatment and prognosis, and missing it, especially in men, may culminate in violent situations that can have legal implications. The developed diagnostic criteria and classifications seem to retain use and should be kept in mind, in the sense that they facilitate making an important diagnosis.

## Background

Erotomania is a psychiatric syndrome characterized by the delusional belief that one is loved by another person, generally of a higher social status [1–3]. This disorder falls under the Persistent Delusional Disorder category in the International Classification of Diseases and Related Health Problemas, 10th revision (ICD-10) [2, 4, 5] and it is classified as a subtype of Delusional Disorder in the Diagnostic and Statistical Manual of Mental Disorders, 5th edition (DSM-5) [6]. Erotic delusions can configure symptoms of any psychotic disorder, and psychiatric manifestations regarding the theme of love have been described since Ancient Greece. Throughout history, several other authors have contributed to the evolution of the concept of Erotomania, but it was the French psychiatrist Gatian de Clérambault (1872–1934) who firstly described the core characteristics of this syndrome (1921). Since then, Erotomania has been known as “de Clérambault’s Syndrome” [1–3, 5, 7, 8].

The main characteristic of de Clérambault’s Syndrome is that the patient (subject), usually a woman, believes she is loved by a man (object). The object must have been the first to fall in love and to make the first advances, the object is more in love than the subject, or the subject may not even return the feelings – it is all about being loved, not loving [1, 2]. De Clérambault also described other components of the syndrome, which he considered to be derived from the main belief [2]. The subject has had very little previous contact with the object of love, which is superior and unattainable in some way, either because he his highly intelligent, incredibly beautiful, famous, belongs to a higher social status, or is married, for example [1, 2]. In some instances, the subject may reject the object of love, however, in most of the cases, he returns the feelings [1]. The subject is convinced that the object cannot be truly happy or complete without her, and if the object is married, the subject believes it is not a valid marriage [1, 2]. The subject believes she watches over the object, that she has indirect conversations with him, and may become bothersome in the object’s life [1, 2]. Despite repeated rejection from the object, the subject keeps believing that the object protects her and that he has difficulties approaching her and cannot return her love for several unexplained reasons. She rationalizes the object’s behavior and interprets his dismissals in a way that confirms that he is in fact madly in love with her. This paradoxical behavior of the object is an essential component of the syndrome that must always be present [1, 2]. De Clérambault described the following stages of evolution of Erotomania: hope, resentment and grudge [1, 2]. The last phase is considered to be the most important. After hoping that the object openly declares his love and by insistently pursuing him, the subject starts feeling humiliated. She may start to hate the object, to become abusive and to claim that the object wronged her, and her delusion of love may even turn into a persecutory delusion [1, 2].

De Clérambault also described two forms of Erotomania: the pure or primary form, and the secondary form [1, 2, 9, 10]. In the pure/primary form, the erotic delusion is the only psychotic manifestation, it is not derived from any other psychiatric or organic illness, hallucinations are absent, the onset is abrupt, and the illness is well defined, having a chronic course. In the secondary form, the onset is gradual, the disease is ill-defined, and the object of love may be substituted by another. Also, the syndrome is associated with other psychiatric illnesses, such as Schizophrenia, Bipolar Disorder and Major Depressive Disorder, and it also may occur combined with Capgras’ Syndrome, Fregoli’s Syndrome and with Folie à Deux. Erotomania may also be secondary to organic conditions, as suggested by more recent reports of cases of Erotomania secondary to head trauma, convulsions, subarachnoid hemorrhage, pregnancy, HIV infection, Cushing’s disease, use of oral contraceptives, premenopause, use of amphetamines, alcohol abuse, mental retardation, Alzheimer’s disease [2, 8, 11].

De Clérambault himself stated that the characteristics described above are rarely present all simultaneously and Erotomania has always been a target of attempts of conceptualization, since there are no specific guidelines for diagnosis [1, 7–9, 12]. Furthermore, the idea that it may be useful to regard Erotomania as an independent syndrome has been questioned to this day. Taylor et al. (1983) defined the following diagnostic criteria: delusional belief that a woman is loved by a specific man, with whom she had had very little previous contact with and that is unattainable in some way, because he is married or, more commonly, because his social position would preclude the development of a relationship. The man watches over the woman, protects or follows her, and the woman remains chaste [12]. There is also mention of the chronic course of the disease. These authors consider there are some grounds to regard Erotomania as a distinct nosological entity [12]. Ellis and Mellsop (1985) adopted the following criteria for the diagnoses of pure/primary Erotomania: a) a delusional conviction of being in amorous communication with another person; b) this person is of much higher rank; c) this other person had been the first to fall in love; d) the other person had been the first to make advances; e) the onset is sudden; f) the object of the amorous delusion remains unchanged; g) the patient provides an explanation for the paradoxical behavior of the loved one; h) the course is chronic; i) hallucinations are absent [7]. In contrast with Taylor et al., after applying the above-mentioned criteria, these authors found no interest in regarding Erotomania as a distinct clinical syndrome [7].

In another attempt to conceptualize Erotomania, Seeman (1978) divided the syndrome in two groups: the fixed group and the recurrent group [8, 9]. In both groups, patients have other psychiatric diagnoses [9]. In the fixed group [9], patients were more psychiatrically ill, and are frequently diagnosed with Schizophrenia. The love object is an ordinary figure of similar status with whom the patient has never had contact, or whom the patient watched from a distance. The delusion does not change and has a chronic course. They are extremely close and dependent on their parents and hold jobs with little responsibility. They see themselves as incapable and have low self-esteem. They are single, timid, withdrawn, sexually inexperienced and have never had a meaningful relationship. In the recurrent group [9], patients are considered to be less psychiatrically ill, and are generally diagnosed with Bipolar Disorder or Personality Disorder. The love object is an important or powerful figure with whom they may have had previous contact with. The patients repeatedly confront the love object, and after being consistently rejected, they accept the impossibility of their love, and go on to repeat the cycle with another love object. They are independent from their parents and have satisfying careers. They have a good self-esteem, are ambitious, competitive and consider themselves to have potential. They have more active love lives and are more sexually experienced.

Erotomania is considered a fairly rare condition, but the exact incidence is not known. It is possible that the incidence is underestimated, given that Erotomania may be classified under broader syndromes. It can present itself from adolescence until old age, and it is not associated with any specific age group, race, culture or socioeconomical status [1, 2, 5]. Interestingly, pure/primary Erotomania is being increasingly reported across a wide variety of settings [5]. Erotomania was thought to be more frequent in females, having once been a disorder exclusively diagnosed in women. There are some case reports about male subjects (around five cases of pure erotomania that the authors are aware of), which predominate over women in forensic samples, and some authors consider male erotomania is far from uncommon [1, 2, 5, 12, 13]. Family association is rare, and there is no evidence of a genetic cause [1, 2, 5]. In general, the love object is of the opposite sex, but there have been reports of homosexual female and male cases [1, 2]. The patient presenting with Erotomania is typically unattractive and sexually inexperienced, never had a meaningful relationship and leads a lonely life (but this is not always the case, as noted above) [14]. Most authors describe a unsatisfactory relationship of the patients with their mothers, while some describe very close and dependent relationships with their parents, sometimes never separating from them, and with the mother being the most important influence [1, 2, 12]. Regarding etiology, there are several proposed explanations for pure/primary Erotomania, ranging from several psychodynamic theories, to neurophysiological data regarding visuospatial functioning deficits, limbic system lesions (particularly if in the temporal lobes), associative deficits, and deficits in cognitive flexibility and in frontal-subcortical functioning. There is also a neurochemical hypothesis that the syndrome may result from a dopamine/serotonin imbalance, and it has been proposed that the interaction between environmental, psychological, pharmacological and physiological factors may trigger Erotomania in a predisposed individual [1, 2, 10]. Pure/primary erotomania is generally considered a chronic and refractory condition with poor prognosis; however, there have been reports of cases with good prognosis [1, 2, 8]. Erotomania secondary to Schizophrenia or Schizoaffective Disorder has a bad prognosis, and erotomania secondary to affective disorders, such as Bipolar Disorder, seems to have a more benign course, with patients having a recurrent pattern of illness and maintaining high levels of functioning [1, 2, 8]. In terms of treatment, anti-psychotic medication has proven useful in reducing the intensity of the delusion and in controlling behavior [1, 2]. Nowadays, risperidone in doses under 6 mg/day is the first line treatment, and pure/primary Erotomania seems to have a better response to neuroleptic treatment than other psychotic disorders [1, 2]. Electroconvulsive therapy seems to show some efficacy when combined with other treatment modalities. Individual psychotherapy is not efficient in these patients, but they may benefit from other family, social and environmental interventions [1, 2, 8, 10]. Some patients may need to be temporarily separated from their love objects, either by hospitalization or imprisonment, and there is evidence suggesting that the separation from the love object may constitute the only truly efficient treatment for Erotomania [1, 2, 8]. Actually, Erotomania may course silently for years and be diagnosed only when the subject stalks or presents violent behavior towards the object. The risk factors for violence in Erotomania are male sex, low socioeconomical status, multiple objects of love and previous history of anti-social behavior [1, 2, 5, 12, 13, 15].

Since descriptions of Erotomania are almost exclusively regarding women, by describing a unique case of Erotomania in a male. we hope to add to literature, and to reflect on the implications of the occurrence of Erotomania in men. By discussing the case in light of the different described clinical pictures, proposed diagnostic criteria and classifications, we hope to contribute to the ongoing attempt to conceptualize this syndrome and to understand the pertinence of considering it an independent nosological entity. Increasing awareness of this illness may also lead to future developments in management and treatment [10].

## Case presentation

Mr. X is a 55-year-old Caucasian male living in a small village in the Inner South of Portugal. He is an only child and lived with his parents his entire life in this village where everyone knows one another. More recently, after his father’s death, he continued living alone with his mother. He graduated from high-school, and afterwards he worked with his parents in a coffee house that they owned. They sold the establishment some years ago, and now he has no employment nor occupation, and sustains himself with the profits of rented properties, having a low socioeconomical status. He spends his days frequenting another coffee house with a very small group of two friends, having no other friends nor social contact.

Mr. X always had a very close relationship with his mother, describing her as his best friend. He had a troublesome relationship with his father. He describes himself as having very low self-esteem and considers himself an unattractive man. He is very vague when asked about previous relationships, mentioning he never had any lasting or significant previous relationships, and he hesitantly reports his first sexual intercourse at the age of 18. He also states that he has a strong desire of having a wife and children, and fears this will never happen because he feels he has no luck with women.

When Mr. X was 51 years old, he started believing that a married lady that frequented the same coffee house that he did, Mrs. A, was in love with him. He was certain of this because of the way that she looked at him and by her gestures, sending him signals, and he returned her love. He approached Mrs. A and had very brief interactions with her, and he found in their short innocent conversations further evidence that she was in love with him. He believed that they were deeply in love, that her marriage was doomed and that she would only be truly happy with him. He was very insistent in approaching her, and Mrs. A rejected further interaction. He started sending her text messages and going to the coffee house at the specific times that he knew she would be there to watch her from afar. Then he started stalking her around the village. His infatuation ceased when Mrs. A confronted and physically assaulted him, and so Mr. X. stopped stalking her. He was certain that this relationship didn’t work because another divorced lady, the owner of the coffee house he spent his days in, Mrs. B, was also in love with him, and was jealous of Mrs. A. He believed that Mrs. B conspired to end his relationship with Mrs. A by saying bad things about him to other people in the coffee house. He found certainty of Mrs. B’s infatuation and jealousy in an episode in which he had followed Mrs. A to another coffee house, and later that evening, when he tried to return to Mrs. B’s coffee house, she closed the door of the establishment earlier specifically to keep him outside, out of spite, letting other people in as usual. Mr. X stated that he considered having a relationship with Mrs. B, had he not been deeply in love with Mrs. A. After this episode, Mr. X continued spending his days in Mrs. B’s coffee house, having little interaction with her. He holds a grudge against Mrs. A, resenting her for not being strong enough to end her marriage and for believing the gossip about him.

Some years later, at the age of 55, shortly after his mother went to live in a nursing home, Mr. X started believing that another married lady that went to the coffee house, Mrs. C, was also in love with him. He believed that everyone in the coffee house was talking about this, conspiring and scheming behind his back to get them together because Mrs. C asked them to. Mr. X. returned Mrs. C’s love, and then he started stalking her around the village. First, he followed her from a distance, but he eventually went to her workplace to ask her out on a date. Mrs. C declined the invitation, and Mr. X believed this was because she was married and was ashamed of admitting she was in love with him in front of her co-workers. Meanwhile, Mr. X started having complaints of insomnia and anxiety. He believed that Mrs. C that was provoking these symptoms using witchcraft, and he also believed she had shrunk his genitals. Mr. X kept on stalking Mrs. C, and 1 week after the new complaints began, he threatened her with a knife, demanding that she undo the spell. He was apprehended by the authorities and was brought to the emergency unit for psychiatric evaluation. He was admitted as an inpatient in the psychiatry service. He was medicated with risperidone 3 mg per day and diazepam 3 mg per day, with no adverse effects, and his persecutory delusion remitted 4 days later. Physical examination, including neurological, was unremarkable and there were no analytical nor imaging relevant findings (normal blood count, renal and liver function, inflammatory markers, normal vitamin B12 and folic acid levels, normal thyroid function, normal parathyroid hormone levels, normal ceruloplasmin levels, negative drug screening, negative serology for HIV, syphilis, hepatitis B and C, normal EKG and no alterations in CT-scan nor MRI). The was no family history of psychiatric illness or of any remarkable medical condition. He did not present any other psychopathology nor history of other symptoms or disorders, so other psychiatric and organic diagnoses were excluded, and the patient was diagnosed with a Persistent Delusional Disorder. He gained insight to his persecutory delusion, but maintained the erotic delusions, and was discharged to follow-up as an outpatient.

Throughout follow-up, the same pharmacological treatment was continued, with good adherence and no observed nor reported adverse effects upon clinical observation and interview. He has not presented further aggressive behavior, he feels better and is satisfied with the medication. He retains the delusional belief that all three ladies are in love with him and are extremely unhappy without him, but these beliefs are less intense. Mr. X is not in love with Mrs. B nor Mrs. C anymore, but remains in love with Mrs. A, and he considers having had the only significant relationship of his life with her. The timeline of this case is summarized in Table 1.

**Table 1:** Timeline

**51 years old**	First and second erotic delusions
**55 years old**	Third erotic delusion
**55 years old, one week before admission as inpatient**	Persecutory delusion
**Day of admission as inpatient**	Aggressive behavior Treatment initiated
**Four-day stay in psychiatric ward**	Remission of persecutory delusion with insight Erotic delusions still present
**Follow-up as outpatient**	Erotic delusions still present but less intense, without further aggressive behavior

## Discussion and conclusions

This case fits the ICD-10 diagnostic criteria for Persistent Delusional Disorder and for DSM-5 Delusional Disorder, Erotomaniac type. Regarding the description made by de Clérambault, our patient believed Mrs. A, Mrs. B and Mrs. C fell in love with him and were the first ones to make advances – Mrs. A looked at him in a special way, Mrs. B tried to sabotage his relationship with Mrs. A because she was jealous, and Mrs. C asked people in the coffee house to pair them up. Both Mrs. A and Mrs. C were unattainable because they were married, and Mrs. B, although divorced, is a business owner, which in a small village, like this one, makes her a known and important figure, even more so because she is the owner of the place where Mr. X spends his days. Our patient returned Mrs. A and Mrs. C’s feelings, and states that he could have also had a relationship with Mrs. B. He believes that at least Mrs. A cannot be truly happy without him because she has a bad marriage. He had little previous interaction with both Mrs. A and Mrs. C, having only seen them in the coffee house. However, once again, because we are talking about a small village, he already knew who Mrs. A and Mrs. C were. Regarding Mrs. B, he also knew her from the coffee house and he saw her every day, but had no close contact with her, only brief client-customer interactions. Mr. X believed his few brief conversations with Mrs. A were much more substantial than they were, and believed he maintained indirect conversation with her by text message and by the signals she sent him. Despite repeated rejection from Mrs. A, Mr. X kept his belief and, and explained this paradoxical behavior with the interference a new love object, Mrs. B. Mr. X stalked Mrs. A and only left her alone after confrontation. Mr. X also explained Mrs. C’s paradoxical rejection with the shame she felt in admitting she was in love with him, he also stalked her, and then he turned to the grudge phase of the syndrome and developed a persecutory delusion regarding Mrs. C. It seems both the delusion regarding Mrs. A and Mrs. C grossly correspond to the description made by de Clérambault. Also, both love object demonstrates paradoxical behavior, which is considered an essential component of the syndrome that must always be present, and a grudge phase is also present in both cases.

We could consider this a case of pure/primary Erotomania in the sense that the symptoms are not secondary to another psychiatric or organic disorder. However, we cannot be certain if the onset was abrupt, and there are multiple love objects and the course is recurrent, which is more typical in the secondary form of the illness. Even so, this case resembles a case described by de Clérambault himself, in which a male patient started with general ideas of reference, then progressed to a more specific love delusion. This patient believed that everyone was conspiring to procure him a wife, he believed to be loved by at least two women at once, and when his beliefs were challenged by direct rejection, he appeared to rapidly give up on them [12]. In regard to Ellis and Mellsop’s (1985) [7] diagnostic criteria for pure/primary erotomania, this case fits all the criteria except criterion f) - the object does not remain unchanged, criterion h) – the course is recurrent and not chronic, and we are not sure about the onset, which is supposed to be sudden to fulfill criterion e) [7].

When looking at Seeman’s (1978) [9] attempt to divide Erotomania in two groups, we can see that our patient presents characteristics from both the fixed and the recurrent group – there are cycles with multiple love objects, with the love object being substituted after repeated rejection like in the recurrent group, and like in the fixed group, Mr. X is single, sexually inexperienced, he has never had a meaningful relationship, he is timid, with low self-esteem, he is extremely dependent and close to his mother, he never had a demanding job and presently has no professional occupation and, with the exception of Mrs. B, his love objects are rather ordinary figures with whom he had previous little contact [9]. Unlike the patients described by this author, Mr. X has no psychiatric diagnosis.

In regard to Taylor et al.’s (1983) [12] criteria, our patient seems to fulfill all of them except the sudden onset, which we are not sure about. This author reports four cases of male Erotomania, and the general profile of the described patients is similar to Mr. X’s case, with the exception that these men all had mood disturbances. These were lonely men who had solitary lives for many years prior to the onset of the delusions. Three of them never had a satisfactory relationship and had no sexual experience. All men had extremely close and dependent relationships with their parents, especially with their mothers. Three of the men had multiple love objects and a recurrent course of illness, and all of them showed aggressive behavior, three of them committing and one of them threatening to commit offenses against people in the context of their disorder, which is in line with the higher prevalence of male patients with Erotomania in forensic samples, as male sex is a risk factor for aggressive behavior [12]. Mr. X also exhibited violent behavior, stalking Mrs. A and Mrs. C and threatening Mrs. C with a knife, and only came in contact with mental health care services as a result of this offense, which is not uncommon for patients with Erotomania. Mr. X presents two other risk factors for violence, namely the presence of multiple love objects and the low socioeconomical status [2]. Mr. X had to be separated from Mrs. C by means of hospitalization, and responded well to first line treatment and is stable, presenting with adequate behavior and with much less intense delusional beliefs.

Mr. X’s case fits de Clérambault description of Erotomania, and the most important components of the syndrome, the paradoxical behavior of the object and the grudge phase, are present in both Mrs. A and Mrs. C’s case. However, and also regarding Ellis and Mellsop’s (1985) [7] criteria for pure/primary Erotomania, although the disorder is not secondary to other illnesses, Mr. X’s case lacks the chronic course with the presence of only one love object, and we are unsure about the sudden onset. Although presenting a course of illness typical of Seeman’s (1978) [9] recurrent group, he presents a profile more typical of the fixed group. Mr. X. finds himself unattractive and is sexually inexperienced, never had a serious relationship and leads a rather isolated life, much like most descriptions of patients with Erotomania [14]. Also, he is incredibly close and dependent of his mother, which is also typical according to some authors [12]. Much alike other Erotomania reports, this case, fits the different criteria and classifications in a heterogeneous way [10]. Interestingly, this case resembles other reports of male Erotomania, where a recurrent course and the presence of multiple love objects is common, also resembling a male case reported by de Clérambault himself [12]. Since these cases tend to be similar, and since male patients are more commonly violent, being more frequently a part of forensic samples, it would be interesting to understand if the syndrome of male Erotomania differs from female Erotomania in other important ways [13], perhaps configuring a somewhat separate subtype of Erotomania. However, there are still not enough reports for us to be able to say that with any certainty.

We can conclude that it seems reasonable to retain the operative concept of Erotomania as a subtype of Persistent Delusional Disorder/Delusional Disorder, since cases fitting the classical descriptions of the syndrome have been reported, and the diagnosis of Erotomania has particular implications in case management, treatment and prognosis – risperidone under 6 mg/day is the first line treatment, to diminish the intensity of the delusions and for behavioral control but, sometimes, the only effective treatment is separation from the love object and risk management, since the patient may become really bothersome and intrusive in the object’s life [14]. Also, missing this diagnosis may culminate in violent situations that may have legal implications, since these patients, especially if male, can present for the first time with violent behavior. We can also conclude that the developed diagnostic criteria and classifications retain use and should be kept in mind, in the sense that they help make clinicians aware of the heterogeneity of the syndrome, and this could facilitate making an important diagnosis that could have serious implications if missed. Increasing awareness of this illness may also lead to future developments in management and treatment [10].

## Data Availability

Not applicable.

## Abbreviations

ICD-10: International Classification of Diseases and Related Health Problems, 10th revision
DSM-5: Diagnostic and Statistical Manual of Mental Disorders, 5th edition
